# Wafer map failure pattern classification using geometric transformation-invariant convolutional neural network

**DOI:** 10.1038/s41598-023-34147-2

**Published:** 2023-05-19

**Authors:** Iljoo Jeong, Soo Young Lee, Keonhyeok Park, Iljeok Kim, Hyunsuk Huh, Seungchul Lee

**Affiliations:** 1 Department of Mechanical Engineering, Pohang University of Science and Technology (POSTECH), Pohang, Republic of Korea; 2 Graduate School of Artificial Intelligence, Pohang University of Science and Technology (POSTECH), Pohang, Republic of Korea; 3 Institute of Convergence Research and Education in Advanced Technology, Yonsei University, 50 Yonsei-ro, Seoul, Republic of Korea

**Keywords:** Computer science, Software, Electrical and electronic engineering, Mechanical engineering

## Abstract

Wafer map defect pattern classification is essential in semiconductor manufacturing processes for increasing production yield and quality by providing key root-cause information. However, manual diagnosis by field experts is difficult in large-scale production situations, and existing deep-learning frameworks require a large quantity of data for learning. To address this, we propose a novel rotation- and flip-invariant method based on the labeling rule that the wafer map defect pattern has no effect on the rotation and flip of labels, achieving class discriminant performance in scarce data situations. The method utilizes a convolutional neural network (CNN) backbone with a Radon transformation and kernel flip to achieve geometrical invariance. The Radon feature serves as a rotation-equivariant bridge for translation-invariant CNNs, while the kernel flip module enables the model to be flip-invariant. We validated our method through extensive qualitative and quantitative experiments. For qualitative analysis, we suggest a multi-branch layer-wise relevance propagation to properly explain the model decision. For quantitative analysis, the superiority of the proposed method was validated with an ablation study. In addition, we verified the generalization performance of the proposed method to rotation and flip invariants for out-of-distribution data using rotation and flip augmented test sets.

## Introduction

Classification of wafer bin map patterns is gaining attention as a critical approach for increasing yield and quality in semiconductor manufacturing processes by allowing root cause analysis (RCA)^1,2^. As integrated circuit (IC) chips, which are composed of electronic circuits that enable desired functions in various electrical products, continuously decrease in size, their manufacturing process becomes more sophisticated, making the cause of defects in the process more difficult to analyze^3^. At a later stage of the semiconductor production process, prior to packaging, different electrical and thermal tests are undertaken to evaluate whether each chip is normal at the wafer die level in binary. Then, defects are displayed on a chip-by-chip basis on the wafer, and this forms a defect pattern. Because this defect pattern is the end outcome of the whole procedure, it is feasible to analyze the correlation between the defect pattern and the process history and details, enabling RCA in the process. Therefore, wafer map defect pattern classification is particularly important in this field because it is strongly tied to improving the quality that the semiconductor industry is aiming for while also raising the production yield.

In addition to the pattern-based defect classification, there has been increasing demand for the automation of the classification process. The wafer map pattern labeling process is directly conducted by experts in the field, which is labor- and cost-intensive, and diagnosis performance varies depending on engineers. Recent research on automated labeling using the wafer map classification model has been conducted due to the data-based classification model’s superior automation capabilities in a variety of sectors. Existing approaches can be classified into two categories depending on the data-driven inference mechanism: (1) machine learning-based and (2) deep learning-based.

Machine learning-based approaches for wafer defect pattern classification utilize a variety of prediction models to extract class discriminative features based on several hand-crafted features derived from the wafer map. Yuan et al.^4^ proposed the classification of spatial defect patterns using support vector clustering and the Bayesian method. Wu et al.^5^ proposed a support vector machine (SVM) based method using a set of Radon and scale-invariant features. He demonstrated that Radon-based features can be used to acquire rotation-equivariant response. Yu and Lu^6^ proposed the use of joint local and non-local linear discriminant analyses for wafer map defect detection and recognition based on multiple features, including geometric and Radon features. Saqlain et al.^7^ proposed a voting ensemble classifier using various features, including Radon features. Various models employing useful features have been actively examined for these methods based on domain knowledge; however, there exists a limitation in terms of the inference performance due to the shallowness of the machine learning-based models.

As the depth of the inference model increases due to the development of computational resources, deep learning-based methods have been actively studied for wafer defect pattern classification because they can automatically learn meaningful features from raw data without expert intervention, enabling improved pattern classification performance. This deep learning-based method follows two steps: First, the deep learning framework is simply applied to the wafer map defect pattern problem; second, practical concerns, such as data scarcity and memory efficiency, are addressed. Regarding the former, early research adopted convolution neural network (CNN) models, which show exceptional performance among deep learning models in image classification, for wafer map classification^8,9^. Kyeong et al.^10^ proposed mixed-type defect patterns in wafer bin maps using multiple CNN models. Yu et al.^11^ proposed two stages for recognizing and classifying wafer map patterns. However, obtaining sufficient clean labeled wafer map data of high quality is often a constraint throughout the manufacturing process; therefore, a model including additional approaches to the traditional CNN model is required. Regarding the latter, several studies have proposed models based on the fact the label remains unaffected by the rotation and flip, according to the predefined labeling rule of the wafer map. Kang et al.^12^ proposed a data augmentation method to learn rotation- and flip-invariant representation through augmention along a discrete angle direction. Kahng et al.^13^ proposed self-supervised learning for pretext-invariant representation, which includes rotation invariance in the data-augmentation context. As a result, it was possible to achieve high classification performance in limited data situations. However, these previously proposed methods have a limitation because they do not directly incorporate rotation and flip invariance into the model architecture, which means that the model ability to recognize these invariances is not specifically built into its design. Instead, these methods rely on data augmentation and additional parameters, which can be inefficient and insufficient for addressing memory efficiency concerns. This has already been noted for rotational variable CNNs in the field of computer vision, as discussed in “Related works”.

In this paper, we propose a novel method for classifying wafer defect patterns that is invariant to rotation and flip. Considering the orientation variations in wafer defect patterns due to manufacturing processes and equipment, achieving rotation and flip invariance becomes crucial for accurate and robust classification. Furthermore, by incorporating these invariances into the classification method, our approach can efficiently extract relevant features from limited data, helping to mitigate data scarcity issues. To achieve rotation and flip invariance, we utilize the equivariant traits of Radon features, a hand-crafted feature previously used in machine learning, within the CNN framework. Moreover, we achieve flip invariance by designing kernels within the network, minimizing the reliance on data augmentation. To validate our model, we conduct both qualitative and quantitative analyses. For qualitative analysis, we introduce the multi-branch layer-wise relevance propagation (multi-branch LRP) method to interpret the model decisions, specifically designed for models with multi-branch structures like our kernel flip module. We demonstrate the individual impact of Radon transformation and kernel flip through both qualitative and quantitative evaluations using an ablation study. We also evaluate our model's unseen generalization performance under rotation and flip augmented dataset.

## Background and preliminaries 

### Related works

CNNs inherently possess a strong capability to learn translation-invariant features through translational weight sharing and pooling operations. However, achieving other forms of spatial invariance, such as rotation and flip, remains a limitation of the CNN framework. Numerous studies have been conducted to address these challenges by (1) augmenting features of an input image with several transformed copies, and (2) encoding desired transformation invariance for the CNN using specific trainable modules within the network.

The former can be broken down into input data augmentation and feature augmentation by the inner filters of the network. In many early studies, the input data were directly augmented for various applications. Laptev et al.^14^ proposed a transformation invariant pooling (TI-pooling) layer taking highly activated transformation-invariant features by max-pooling to the fully connected layer, extracted over a weight-shared CNN for each input based on the rotationally augmented training dataset. Cheng et al.^15^ proposed a similar method, rotation invariant CNN (RICNN), which trains existing CNNs by rotationally augmenting training samples for the object detection task. Cheng et al.^16^ proposed a Rotation-invariant and fisher discriminative CNN (RIFD-CNN), also using the data augmentation strategy as RICNNs but adding a Fisher-discriminatory layer. However, directly augmenting input data has a critical limitation that fundamentally requires higher memory size and network capacity to obtain more generalizable rotation. Because of this, feature augmentation by internal filters of the network has lately gained considerable attention in a variety of methods. Dieleman et al.^17^ proposed the multiple branch structure of a CNN for extracting different viewpoints for each augmented image. Then, Dieleman^18^ extended this concept by performing various operations on cyclic symmetries. Cohen et al.^19^ proposed a group-equivariant CNN based on group theory, utilizing a symmetry group and pooling operation on the group. Marcos et al.^20^ suggested explicitly incorporating the rotation invariance method into the model by associating the weights of groups of filters with various rotated copies of the group’s canonical filter. Gao et al.^21^ proposed a set of kernel rotation and flip methods for achieving rotation and flip invariance in a CNN. In summary, the feature augmentation method follows the structure of sampling multiple branches for data variation within the network, and the main limitation of this is the trade-off relationship between generalizing the data variation and the number of branches.

The second work is the utilization of certain trainable modules inside a CNN to encode required transformation invariance for the CNN. Worrall et al.^22^ proposed a harmonic networks that achieves rotation invariance by replacing regular CNN filters with circular harmonics, thus returning a maximal response and orientation. Jaderberg et al.^23^ proposed the spatial transformer network (STN), which uses learnable modules, explicitly allowing the spatial manipulation of input data to reduce pose variations in subsequent layers within the network. Esteves et al.^24^ suggested a polar transformer network (PTN), which is an extended version of STN combining canonical coordinate representations. Dai et al.^25^ proposed a deformable CNN with deformable convolution and RoI pooling based on the idea of augmenting the spatial sampling locations in the modules. These works have constraints in that they not only require additional trainable parameters for additional modules but also require a complex structure to adapt to a CNN.

In this study, we propose a novel rotation and flip invariant CNN approach for classifying wafer map defect patterns, taking into consideration the challenge of data scarcity. To achieve this, we suggest incorporating handcrafted features into a deep learning framework. Specifically, we utilize the rotation-equivariant property of the Radon feature, a commonly used hand-crafted feature in previous machine learning context for wafer classification task, to obtain rotation invariance in the CNN framework. Furthermore, we achieve flip invariance by introducing a kernel flip module with only a two-branched structure, which learns the data variation of flipped copies produced by each branch. It is worth noting that our method achieves flip invariance in all directions by securing it in combination with rotation invariance, utilizing the rotation-equivariant feature and minimal branches of the flipped kernel. This approach allows for more compact and efficient representations, potentially leading to better performance and reduced training times compared to data augmentation-based methods.

### Equivariance and invariance

To facilitate understanding of the problem statement, it is essential to first comprehend the concepts of equivariance and invariance. Given a mapping function $\Phi$, an input $X$ from a set of inputs {${X}_{i}$}, and a group $G$, we call $\Phi$ equivariant under ${T}_{1}\in G$ if the transformation of the input is related to a transformation ${T}_{2}\in G$ of the output, as stated in Eq. (1). Conversely, $\Phi$ is invariant under $T$ if it is independent of the transformation relationship in the output domain, as expressed in Eq. (2).

1 $$\Phi ({T}_{1}({X}_{i})) = {T}_{2}(\Phi ({X}_{i}))$$

2 $$\Phi ({X}_{i}) = \Phi (T({X}_{i}))$$

### Problem formulation

To clearly explain the proposed mechanism of obtaining rotation and flip invariance, we formulated the principle of the proposed approach including Radon transform, kernel flip, and CNN backbone module. The wafer defect pattern image data and its label set exist as $\left({X}_{i}, {y}_{i}\right)$, geometrical transformations are denoted as translation: ${T}_{T}$ rotation: ${T}_{R}$ , flip: ${T}_{F}$, and each group of each transformation is denoted as ${G}_{T}, {G}_{R},$ and ${G}_{F}$. The labeling rule function $({\Phi }_{label}$) is given according to Eq. (3) when $T={T}_{R}\cdot {T}_{F}={T}_{F}{\cdot T}_{R}$ in ${G}_{R}\cup {G}_{F}$, where ${T}_{R}\cdot {T}_{F}$ represents function composition of ${T}_{R}$ and ${T}_{F}$, and our objective is to build a model that approximates this function:

3 $${\Phi }_{label}\left(T\left({X}_{i}\right)\right)= {\Phi }_{label}\left({X}_{i}\right)={y}_{i}$$

The CNN model (${\Phi }_{CNN})$ we use for label inference has the inherent ability to learn translation-invariant features, exhibiting the following characteristics:

4 $${\Phi }_{CNN}\left({X}_{i}\right)={\Phi }_{CNN}\left({T}_{T}\left({X}_{i}\right)\right)= \overline{{y }_{i}}$$

However, the CNN model is not rotation-invariant:

5 $${\Phi }_{CNN}\left({T}_{R}\cdot {X}_{i}\right)\ne {\Phi }_{CNN}\left({X}_{i}\right)$$

To provide some context for Eq. (5), let ${T}_{R}\cdot {X}_{i}$ represent the application of the rotation transformation ${T}_{R}$ to the input ${X}_{i}$. With this understanding, we can now explain that our model uses the rotation-equivariant mapping function Radon transform $({\Phi }_{Radon})$ as an intermediate step to address the lack of rotation invariance in the CNN model.

6 $${\Phi }_{Radon}\left({T}_{R}\cdot {X}_{i}\right)={T}_{T}\left({\Phi }_{Radon}\left({X}_{i}\right)\right)$$

As a result, we have:

7 $${\Phi }_{CNN}\left({\Phi }_{Radon}\left({T}_{R}\cdot {X}_{i}\right)\right)={\Phi }_{CNN}({T}_{T}({\Phi }_{Radon}\left({X}_{i})\right)={\Phi }_{CNN}\left({\Phi }_{Radon}\left({X}_{i}\right)\right)$$

For our proposed model, we aim to achieve both rotation and flip invariance. To address the lack of flip-invariance, we incorporate the kernel flip (KF) module into the CNN architecture:

8 $${\Phi }_{CNN+KF}\left({T}_{F}{{(\Phi }_{Radon}(X}_{i}))\right)= {\Phi }_{CNN+KF}\left({{\Phi }_{Radon}(X}_{i})\right)$$

The flip symmetry of the wafer map is preserved here by changing the flip axis by $\uppi$/2 to account for the Radon feature effect:

9 $${\Phi }_{Radon}\left({T}_{F}({X}_{i})\right)={T}_{F^{\prime}}\left({\Phi }_{Radon}\left({X}_{i}\right)\right)$$

By combining the rotation and flip transformations, our model can inherently account for all possible flip orientations. According to group theory^26^, the union of the rotation and flip groups remains the same regardless of the orientation of the flip axis:

10 $${G}_{R}\left(X\right)\cup {G}_{F}\left(X\right)= {G}_{R}\left(X\right)\cup {G}_{{F}^{^{\prime}}}\left(X\right)= {G}_{F^{\prime\prime}}(X)$$

As a result, our model can effectively extract rotation and flip invariance, accounting for all possible rotation and flip transformations, while employing the minimal number of flipped kernel branches. Our proposed method is described in detail in the following section.

## Methodology

### Proposed framework

**Figure 1:**
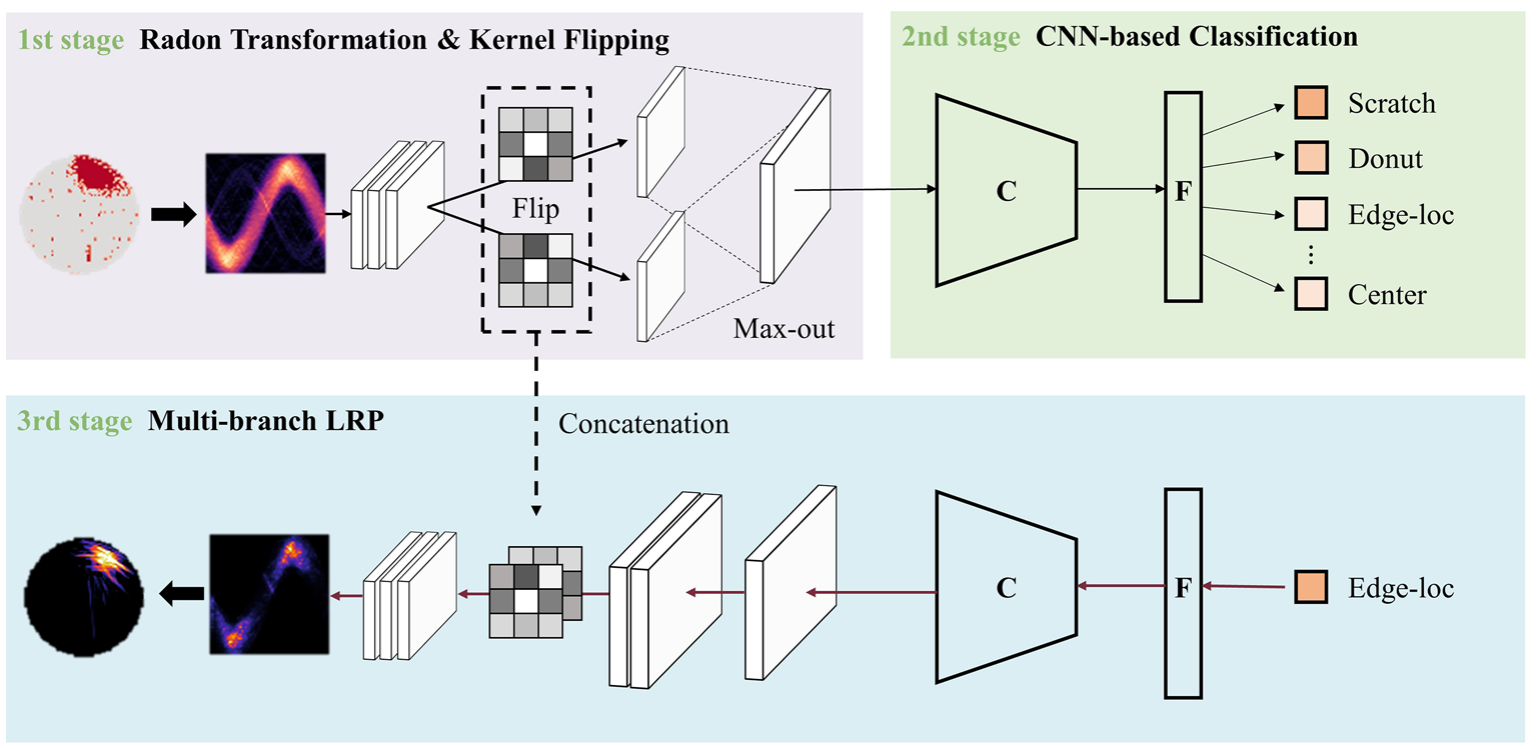
Overview of our method. Upper: the proposed model architecture comprises a Radon transformation and kernel flipping module for acquiring rotation and flip equivariant features, followed by a translation invariant back-bone CNN-based classifier. Lower: the multi-branch LRP method for multi-branch structure induced by kernel flipping, which is used for interpreting model predictions. Wafer map and heatmap images were visualized using Python 3.8.4 and the released WM-811K wafer dataset, available at http://mirlab.org/dataSet/public/. Radon and inverse Radon transforms were performed with the scikit-image library version 0.20.0, while the LRP heatmap was obtained using our proposed multi-branch LRP method.

The proposed rotation- and flip-invariant representation learning method comprises two main modules and a CNN backbone, as illustrated in Fig. 1. Initially, the Radon rotation-invariant module transforms wafer maps into tomography images, converting rotation to translation. Subsequently, a flipped feature set is obtained through two branches of kernel flip operations. By employing the max-out operation on the highly-activated features among the pair of flipped feature sets, the backbone CNN, often referred to as translation-invariant due to its capability of acquiring translation-invariant features, learns a discriminative representation that captures the wafer label characteristics through rotation equivariant and flip equivariant features.

### Radon transformation

Our proposed method adopts the Radon feature as input representation due to its rotation-equivariant characteristic with respect to the wafer map. Radon transformation is a method to acquire sinusoidal tomography ${P}_{\theta }\left(r\right)$ by projection image for rotation $\theta$. The Radon transform is a forward projection to obtain tomography ${P}_{\theta }\left(r\right)$. When f(x,y) is an original image, the Radon transform function is given as,

11 $$r= xcos\theta +ysin\theta$$

12 $${P}_{\theta }\left(r\right)= \sum_{x=1}^{m}\sum_{y=1}^{n}f(x,y)\delta (xcos\theta +ysin\theta -r)$$

**Figure 2:**
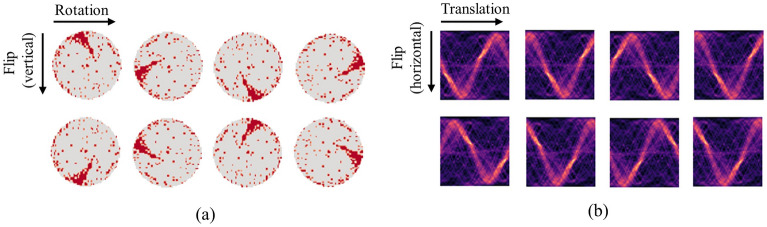
(**a**) Illustration of rotation and flip examples for the Edge-loc class wafer maps from WM-811k and (**b**) the corresponding Radon transforms for each wafer map. All images in this figure were visualized using Python 3.8.4. The Radon transformations were performed using the scikit-image library version 0.20.0.

The above projection converts the original image’s rotation impact to a translation of the Radon feature. By comparing the first rows of Fig. 2a, b, we can recognize that the original wafer map’s rotation corresponds to the Radon feature’s translation. As a result, the Radon transform functions as a rotation-equivalent bridge, enabling the use of a translation-invariant CNN backbone model to obtain rotation-invariant representation. Additionally, by comparing the second rows of Fig. 2a, b, we can see that the vertical flip on the wafer map corresponds to a horizontal flip on the Radon feature. This implies that the flip equivariance of the Radon feature is inherently guaranteed to be flip equivariance for the wafer map, considering the $\uppi /2$ change in the flip axis.

### Kernel flip

Kernel flip modules aim to learn the flip equivariance through generated flipped copies of input features with multiple flip versions of kernels. For our proposed method, we use only two branches of flipped kernels: the original one and the single-axis flipped one. When the processed Radon feature, after passing through several layers, is input into this module, weight-sharing flipped kernels output a flipped feature set without increasing the number of trainable parameters, ensuring model efficiency. By learning this flip variation on Radon-based features, our model is capable of acquiring flip equivariant properties in addition to rotation equivariance. As the main consideration for our narrow branch structure, as described in “Related works”, there exists a trade-off between generalization performance and the number of branches; therefore, the only additional single branch may lead to a weak flip-equivariant performance. However, as described in “Problem formulation”, obtaining flip equivariance on a rotation-equivariant representation corresponds to all directions of flip equivariance. After generating a flipped feature set, the max-out module then takes the most active features element-by-element to pass to the CNN classifier module while aiming at efficient memory flow inside the network and the drop-out effect. Finally, the obtained rotation and flip equivariant features from the Radon and kernel flip modules make it possible to learn the rotation and flip invariance with the CNN classifier.

### Multi-branch LRP

In this study, we adopted the LRP to evaluate our method in a qualitative manner not only to recognize the effect on inference based on the Radon feature in accordance with the original wafer map-based prediction but also to verify that our proposed model works as intended. The LRP is primarily used to comprehend the model inference using an interpretability-based approach to deep learning-based models. Based on the deep Taylor decomposition method described by Eq. (13), the relevance score can be obtained by output prediction, where *a* is a root point of the Taylor series and $\epsilon$ is a substituted term for the Taylor series’ higher-order polynomial terms. By sequentially repeating the relevance propagation to previous layers, the input layer’s relevance scores can finally be obtained.

13 $$f\left(x\right)=f\left(a\right)+ \sum_{i=1}^{d}\partial f/\partial {x}_{i}{\vert}_{x=a}\left(x-a\right)+\epsilon = {\sum }_{i}{R}_{i}$$

To apply this technique to our model, there is a structural consideration that it is difficult to propagate the relevance score as-is because our model is a multi-branch model. To the best of our knowledge, the LRP method has not been used in a complicated structure such as a multi-branch CNN before. Herein, we propose a novel LRP method for the multi-branch structure, as depicted in Fig. 1. When the relevance score has arrived at the kernel flipping modules, two relevance scores are generated after passing each kernel. The propagation of the separated relevance score provides multiple relevance scores that are unrelated to the model judgment grounds at the input layer. To solve this structural problem, we concatenate both relevance scores and both kernels by channel axis. Then, we propagate the relevance through the concatenated relevance feature and kernel to generate a combined relevance score.

## Results and discussion

### Experiment

#### Data description

In general, wafer map patterns are categorized into seven classes based on their cluster position and shape, which has specific process conditions and effects^27^: center, donut, edge-loc, ring, loc, scratch, and random. For example, the center type has the effect of problems in the plasma area^28^ or thin-film deposition, and the edge-loc type has the same effect as uneven heating during the diffusion process. Therefore, it has been considered an important task to classify them and determine the state of the process so that the cause of process deterioration can be estimated. Existing machine learning-based wafer sorting tasks have mainly been researched under two scenarios: individual fab data and open data^27^, each with pros and cons. Using private data is advantageous for optimizing the problem at hand, but methodological generalizations are difficult. However, publicly available data are easier to compare with other methods, implying that the method’s generalization could be claimed; hence, it is preferable to utilize it for verification.

**Figure 3:**
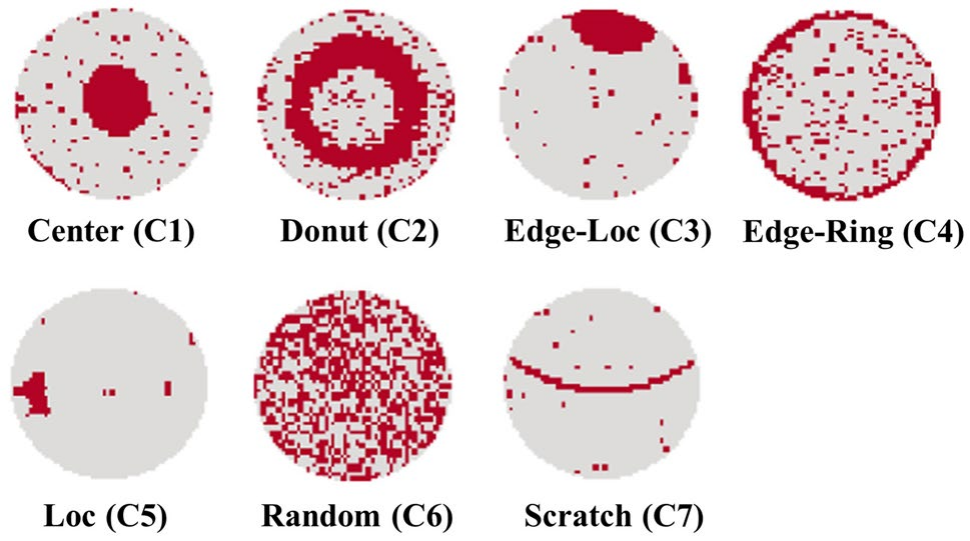
Used wafer patterns of WM-811K for this study. Near-full and none patterns are excluded, as described in “Experimental setup”.

The real-world fab data WM-811K has frequently been used in wafer classification tasks via machine and deep learning^29^. For data representation, each wafer map is formed as a 2D image of varying sizes. As shown in Fig. 3, WM-811K contains a total of nine classes, including the seven aforementioned classes and additional near-full and none classes, with a total amount of 172,950. Among them, there are 25,519 labeled data, which is only approximately 14.8% of the data. Additionally, as shown in Table 1, it has a highly imbalanced data distribution, i.e., near-full class accounts for only 0.1%. The appropriate data processing for the evaluation is addressed in “Experimental setup”.

**Table 1:** Data distribution of WM-811K.

Class	Quantity	Fraction (%)
Center (C1)	4294	2.5
Donut (C2)	555	0.3
Edge-loc (C3)	5189	3.0
Edge-ring (C4)	9680	5.6
Loc (C5)	3593	2.1
Random (C6)	886	0.5
Scratch (C7)	1193	0.7
Near-full	149	0.1
None	147,431	85.2

#### Experimental setup

To assess our proposed method effectiveness, we utilized the seven typical classes from WM-811K as indicated in Fig. 3, with setting balanced data distributions for each class. Previous researches on wafer map pattern classification using WM-811K can be classified into two categories. The first case uses nine classes, while the second only takes seven or eight classes, depending on whether it contains the none or near-full classes. Mohamed et al.^30^ highlighted the negative effects of using the none class, as it can impact both model training and performance analysis for several reasons. Thus, we followed the latter approach by taking seven classes excluding ‘Near-full’ and ‘None’ classes to focus on addressing data scarcity, aside from the data imbalance problem. Then, we sub-sampled train and test datasets for the seven classes with a small dataset ranging from 100 to 6,400 with a balanced data size for each class. To preprocess the data, we first resized the wafer map to (64, 64), and removed the wafer map background, retaining only the defect points due to varying wafer map sizes, which can lead to slightly different shapes on the sides after resizing, thus affecting model training negatively.

To comparatively evaluate the proposed model via an ablation study, we established four comparative models. The first, a baseline model, utilized the wafer map as input to the baseline network, as detailed in Table 2. The second model, the Radon model, took the Radon transformation before inputting the wafer map into the same baseline network. The third model, the kernel flip model, had a two-branched kernel flip module within the baseline network and used the wafer map as input. Lastly, the proposed model incorporated both the Radon transformation and the kernel flip module onto the baseline model which is also detailed in Table 2.

**Table 2:** Baseline and proposed models for comparative analysis.

Layer	Baseline		Proposed	
	Activation	Output shape	Activation	Output shape
Input		64 × 64 × 1		64 × 64 × 1
Radon transform				64 × 64 × 1
Convolution	ReLU	32 × 32 × 16	ReLU	32 × 32 × 16
Batch norm				
Max pool				
Convolution	ReLU	16 × 16 × 64	ReLU	16 × 16 × 64 × 2 (weight-shared kernel flip)
Batch norm				
Max pool				
Max out				16 × 16 × 64
Convolution	ReLU	8 × 8 × 128	ReLU	8 × 8 × 128
Batch norm				
Max pool				
Convolution	ReLU	4 × 4 × 256	ReLU	4 × 4 × 256
Batch norm				
Max pool				
Fully-connected	ReLU	256	ReLU	256
Batch norm				
Fully-connected	ReLU	128	ReLU	128
Batch norm				
Fully-connected		7		7

In the experiments, the initial learning rate was set to 0.0003, and the Adam optimizer was used for updating the model weights. The learning rate decay was used for every epoch with a decay rate of 0.99. The training steps were stopped early when the validation loss did not decrease for 30 epochs to prevent overfitting. The loss function used was the Cross Entropy Loss, which is suitable for classification tasks. Each experiment was repeated 20 times using different random seeds. The results are reported as the average and standard deviation of all the repeated measurements.

#### Evaluation strategy

To evaluate the performance of our proposed method, we conducted both quantitative and qualitative analyses. Firstly, we performed a qualitative analysis using the LRP method to verify the adequacy of our proposed method. Specifically, we visually examined the LRP heat maps to analyze how the model focuses on different parts of the wafer map to make decisions. Additionally, we verified the effect of rotating and flipping the original wafer map on the proposed model inference by assessing how these transformations affect the model attention to the wafer map. Throughout these experiments, we compared the qualitative performance of the baseline and proposed methods. As the LRP heatmap for the proposed method is based on Radon features, direct comparison with the baseline was difficult. Thus, we applied an inverse Radon transform to the relevance scores obtained from Radon feature-based inference, using the projection-slice theorem to verify the consistency between the original wafer map and Radon feature-based inference. This allowed us to compare the proposed method with the baseline.

Secondly, we conducted a quantitative analysis to evaluate the performance of the proposed model. Initially, we performed an ablation study to verify the validity of the proposed method by analyzing the effect of each module on the overall performance of both the entire and sub-classes. In addition, we assessed the impact of rotation and flip on the proposed model performance for each class using the confusion matrix. The degree of variation for rotation and flip differs depending on the wafer map pattern, with some classes exhibiting insignificant variation while others display wide variation. For instance, the center and donut classes contain uniformly defective points in all directions, resulting in insignificant variation for rotation and flip, while the scratch class has a wide variation for flip and rotation since it exists in curved or straight line forms independent of direction and location.

Lastly, to validate the generalization performance of our model, we conducted a thorough comparison of the performance of the proposed model and comparative models on the original test set and an unseen (out-of-distribution) augmented test set. Specifically, we evaluated the ability of the models to generalize to unseen distributions for rotation and flip transformations. While the original test set can be considered unseen as it was not used in training, it was still limited to the distribution within the original dataset. To assess the proposed model robustness to generalization, we generated a dataset by directly rotating and flipping the test set to extend beyond the distribution of the original dataset. The rotation augmented test set included 90°, 180°, and 270° rotationally augmented test sets, while the flip augmented test set included horizontally and vertically flipped test sets. We then integrated the two augmentation methods for rotation and flip. It is important to note that the augmented test set did not include the original test set. This comparison allowed us to confirm the validity of the proposed model architecture and verify its robustness to unseen situations.

### Qualitative analysis

#### Radon transform-based classification

**Figure 4:**
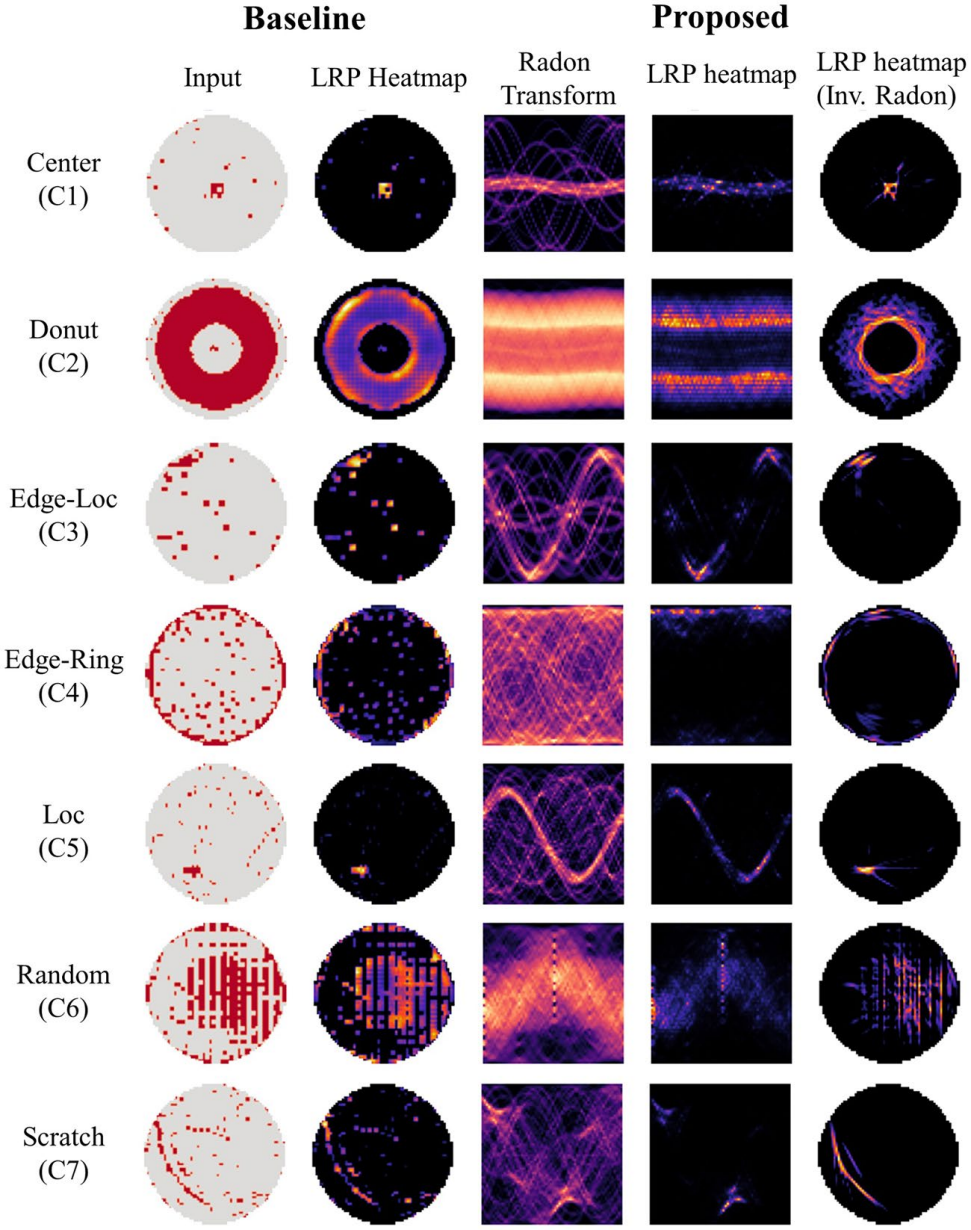
Layer-wise relevance heatmap analysis of the baseline and proposed methods for all classes, with models trained on a train set of size 6400 samples. The first and third columns correspond to the input for the Baseline and Proposed models, respectively. The second and fourth columns depict the LRP interpretation results for the model decisions. The fifth column displays the inverse Radon transform results of the proposed method's LRP outcomes, which are represented to match the original wafer map's shape. All images in this figure were visualized using Python 3.8.4. Radon and inverse Radon transforms were performed with the scikit-image library version 0.20.0, while the LRP heatmap was obtained using our proposed multi-branch LRP method.

To begin, we confirm how the model decision is made for label classification with the obtained LRP heatmaps. Figure 4 compares the baseline model for each class to the proposed model’s relevance score. By examining the second column, it is clear that the baseline model is primarily concerned with the visual pattern represented on the wafer map. Meanwhile, due to the difficulty of directly interpreting the Radon model decision, it was compared using the transformed relevance by inverse Radon transform, as depicted in the fifth column. As a result, it was determined that the proposed model also corresponds to the defect pattern on the wafer map. This is a significant finding because it demonstrates that the shape information contained in the wafer map is retained even when the model is evaluated solely based on the Radon feature. Moreover, by comparing the prediction outcomes, it is evident that the proposed model focuses exclusively on the primary defect location, which explains the higher classification performance. In particular, The results show that for classes such as C3 and C7, the proposed model pays more attention to the location of clear patterns compared to the baseline. This observation is consistent with the fact that C3, C5, and C7 have a wide range of variations in rotation and flip transformations, making it difficult for the baseline model to learn class-discriminative features. In contrast, the proposed model shows robust learning with regards to rotation and flip transformations, which could be the reason behind the observed performance improvement. This finding provides evidence that the proposed method is effective in learning more robust and discriminative features in the presence of diverse image transformations, which can be especially useful for challenging real-world scenarios.

#### Rotation and flip invariant classification

**Figure 5:**
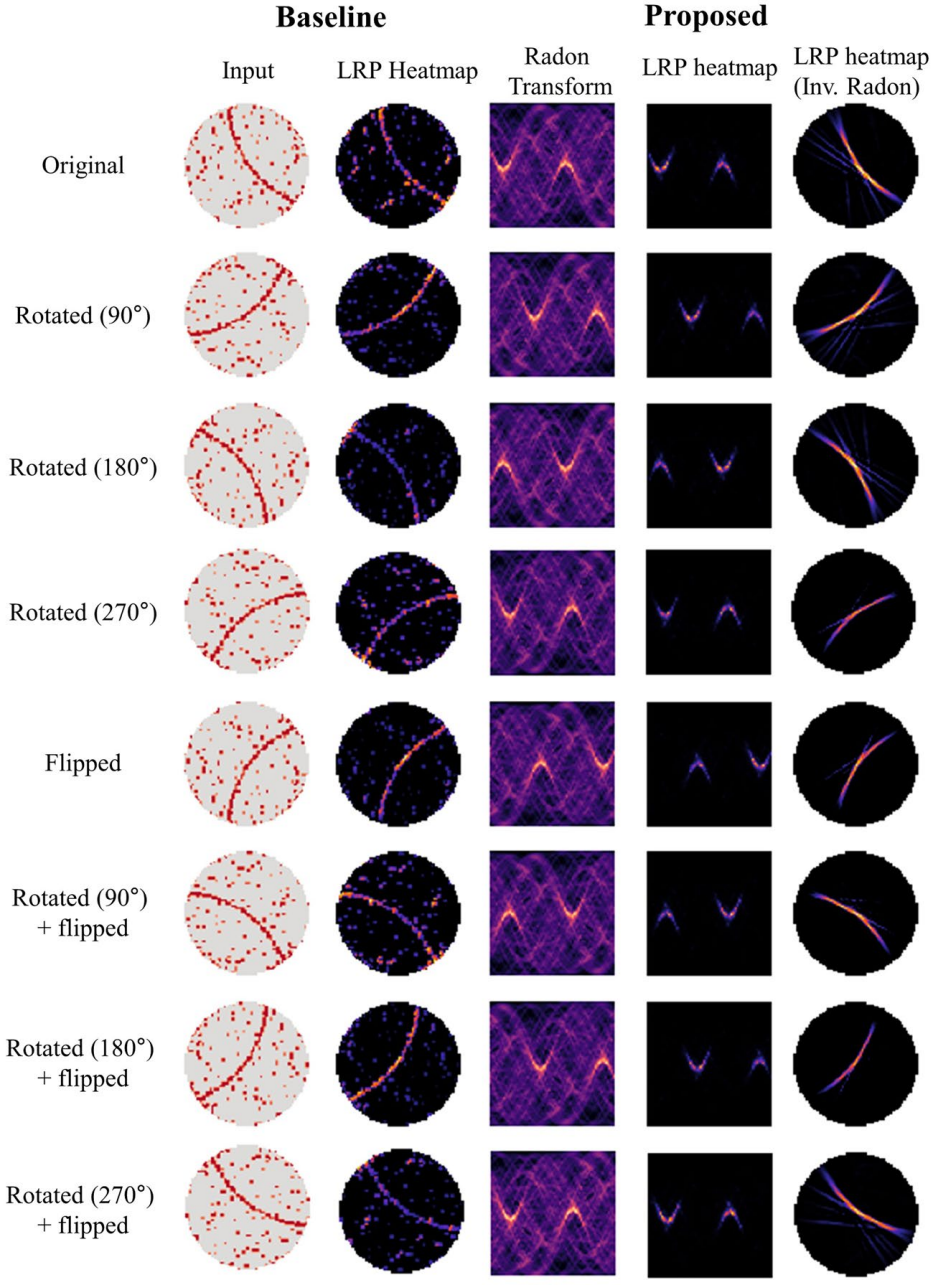
Layer-wise relevance heatmap analysis of the baseline and proposed methods for rotated or flipped test wafer maps, with models trained on a train set of size 6400 samples. The first and third columns correspond to the input for the Baseline and Proposed models, respectively. The second and fourth columns depict the LRP interpretation results for the model decisions. The fifth column displays the inverse Radon transform results of the proposed method's LRP outcomes, which are represented to match the original wafer map's shape. All images in this figure were visualized using Python 3.8.4. Radon and inverse Radon transforms were performed with the scikit-image library version 0.20.0, while the LRP heatmap was obtained using our proposed multi-branch LRP method.

Figure 5 compares the relevance scores of the baseline and proposed models while rotating and flipping the test set by the multi-branch LRP method. The wafer map and Radon feature rows 1–4 exhibit that rotation of the wafer map acts as a translation of the Radon feature, and rows 5–8 demonstrate that vertical flipping of the wafer map acts as horizontal flipping of the Radon feature. Based on the LRP heatmap obtained by the proposed model, the activated region is translated horizontally for the rotated wafer map and similarly for the vertically flipped wafer map. Additionally, by performing an inverse Radon transformation on the proposed model’s heat map, it was confirmed that the relevance score indicates the defect pattern of the original wafer map. As a result, the proposed model’s Radon transform-based kernel flipping method significantly contributes to obtaining rotation and flip invariance for wafer pattern classification.

Another notable point is that whenever the original wafer map is rotated and flipped, the relevance score of the baseline model pays attention to various different positions, but the proposed model focuses more on the defect points of the original wafer map. This indicates that the proposed model has high robustness classification performance for the input wafer rotation and flip variations, which is also the reason why it shows improved classification performance for the original and augmented test sets, as discussed later in “Quantitative analysis”.

### Quantitative analysis

#### Classification performance comparison

**Figure 6:**
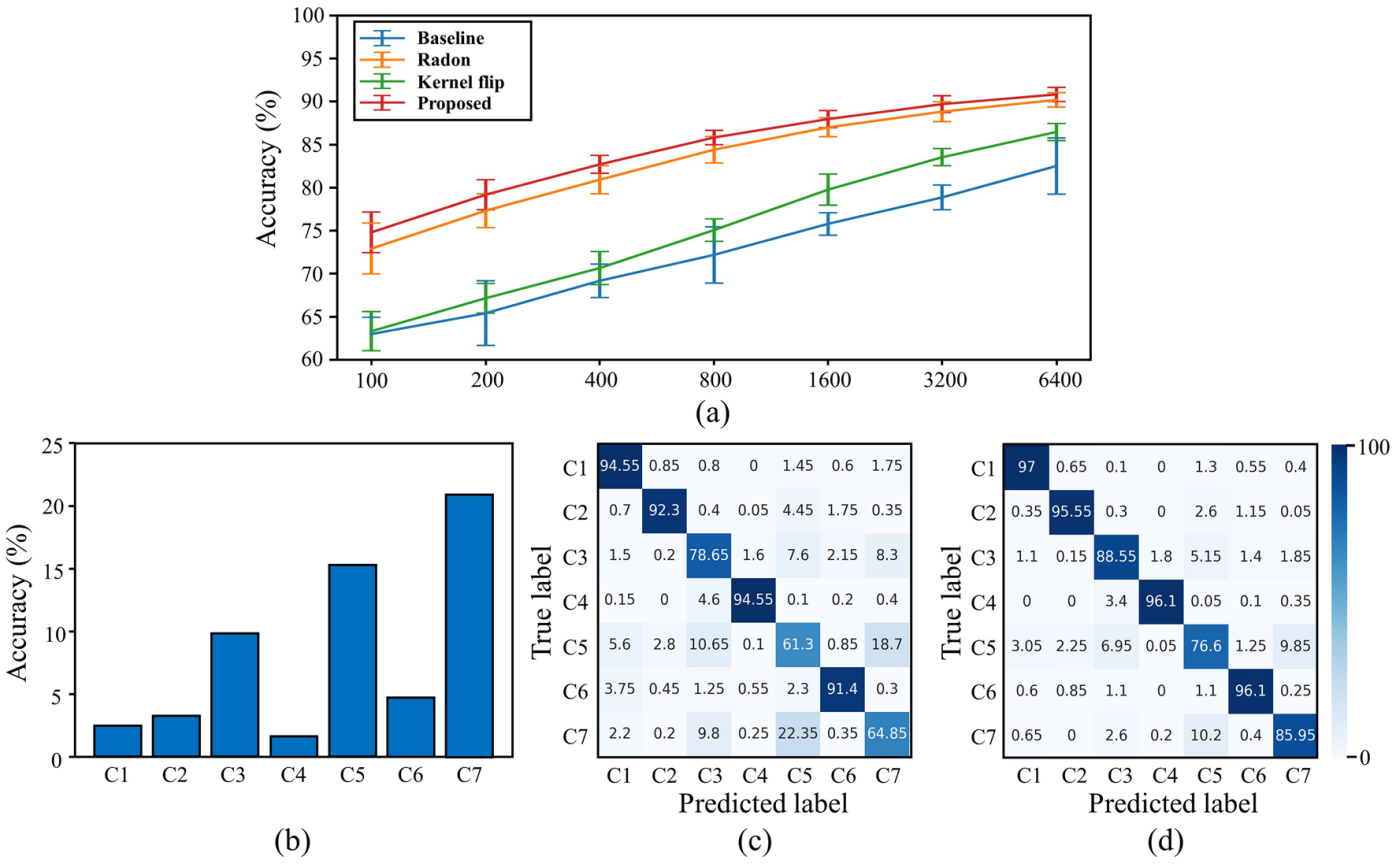
(**a**) Comparison of classification accuracy between comparative models for various train set sizes, (**b**) accuracy gap for each class between the baseline and proposed methods, (**c**) confusion matrix of the baseline model, and (**d**) confusion matrix of the proposed model for a train set of size 6400.

Figure 6a and Table 3 present a comparison of the classification accuracy of the comparative models for various train set settings. The Radon and kernel flip models, as well as the proposed model, exhibit higher classification accuracy than the baseline model. Notably, the Radon model performs better than the kernel flip model, indicating that the wafer map patterns exhibit more variation for rotation than for flip. Of all the methods, the proposed model achieves the highest performance, indicating that invariance is ensured for both rotation and flip.

**Table 3:** Comparison of classification accuracy (%) between comparative models for various train set sizes.

Model	Train set size						
	100	200	400	800	1600	3200	6400
Baseline	63.00 $\pm$ 1.93	65.41 $\pm$ 3.75	69.19 $\pm$ 1.93	72.19 $\pm$ 3.28	75.80 $\pm$ 1.31	78.87 $\pm$ 1.42	82.51 $\pm$ 3.29
Kernel flip	63.33 $\pm$ 2.27	67.16 $\pm$ 1.72	70.66 $\pm$ 1.91	75.07 $\pm$ 1.29	79.77 $\pm$ 1.81	83.54 $\pm$ 0.99	86.48 $\pm$ 0.99
Radon	72.92 $\pm$ 2.96	77.33 $\pm$ 1.95	80.93 $\pm$ 1.64	84.41 $\pm$ 1.56	87.01 $\pm$ 1.10	88.84 $\pm$ 1.15	90.20 $\pm$ 0.83
Proposed	74.81 $\pm$ 2.37	79.19 $\pm$ 1.73	82.71 $\pm$ 1.04	85.83 $\pm$ 0.82	87.97 $\pm$ 1.01	89.71 $\pm$ 0.98	90.84 $\pm$ 0.81

Figure 6b–d presents a comparison of the baseline and proposed models in terms of class accuracy. Figure 6b shows the difference between class accuracy, which is a diagonal element of the confusion matrix (Fig. 6c, d). Figure 6b indicates that the proposed model has a higher accuracy for all classes than the baseline model. In particular, C3 (edge-loc), C5 (loc), C6 (random), and C7 (scratch) are significantly increased among all classes. This trend is matched with the fact that this class has considerably more rotation and flip variance than the other classes. Therefore, it can be confirmed that the high accuracy of the proposed model is derived from the rotation and flip invariance.

#### Generalized classification performance for unseen rotated and flipped test set

Table 4 compares the classification accuracy of comparative models for augmented test sets. In rows 1–2, the baseline and kernel flip models are evaluated under the flip augmented test set. In rows 3–4, the baseline and Radon models are evaluated under the rotation augmented test set. In rows 5–6, the baseline and proposed models are evaluated under the rotation and flip augmented test set. For all cases, comparative models score higher accuracy than the baseline model. This means that the proposed model and its ablation models work as rotation- or flip-invariantly to the unseen augmented situation for rotation or flip.

**Table 4:** Comparison of classification accuracy (%) between comparative models for unseen augmented test sets.

Model	Augmented	Train set size						
		100	200	400	800	1,600	3,200	6,400
Baseline	Flipped	62.51 $\pm$ 2.10	64.11 $\pm$ 3.73	68.20 $\pm$ 1.79	70.46 $\pm$ 2.94	73.49 $\pm$ 1.17	77.02 $\pm$ 1.13	79.90 $\pm$ 2.93
Kernel flip	Flipped	62.13 $\pm$ 2.26	65.28 $\pm$ 1.36	69.17 $\pm$ 1.32	73.36 $\pm$ 0.91	77.65 $\pm$ 1.36	82.03 $\pm$ 1.08	84.93 $\pm$ 1.05
Baseline	Rotated	61.78 $\pm$ 1.93	62.75 $\pm$ 4.09	67.51 $\pm$ 2.08	69.14 $\pm$ 2.64	71.85 $\pm$ 1.39	75.40 $\pm$ 1.49	77.79 $\pm$ 3.11
Radon	Rotated	71.73 $\pm$ 2.73	75.63 ± 1.85	79.68 $\pm$ 1.54	82.76 $\pm$ 1.14	84.94 $\pm$ 0.82	87.01 $\pm$ 0.81	87.80 $\pm$ 1.03
Baseline	Rotated + flipped	62.01 $\pm$ 2.08	63.20 $\pm$ 3.93	67.60 $\pm$ 1.90	69.49 $\pm\,\, 2.61$	72.24 $\pm$ 1.16	75.92 $\pm$ 1.27	78.25 $\pm$ 2.95
Proposed	Rotated + flipped	73.83 $\pm$ 1.98	77.32 $\pm$ 1.55	81.28 $\pm$ 1.14	84.35 $\pm$ 0.92	85.92 $\pm$ 0.76	87.89 $\pm$ 0.64	88.75 $\pm$ 0.59

**Figure 7:**
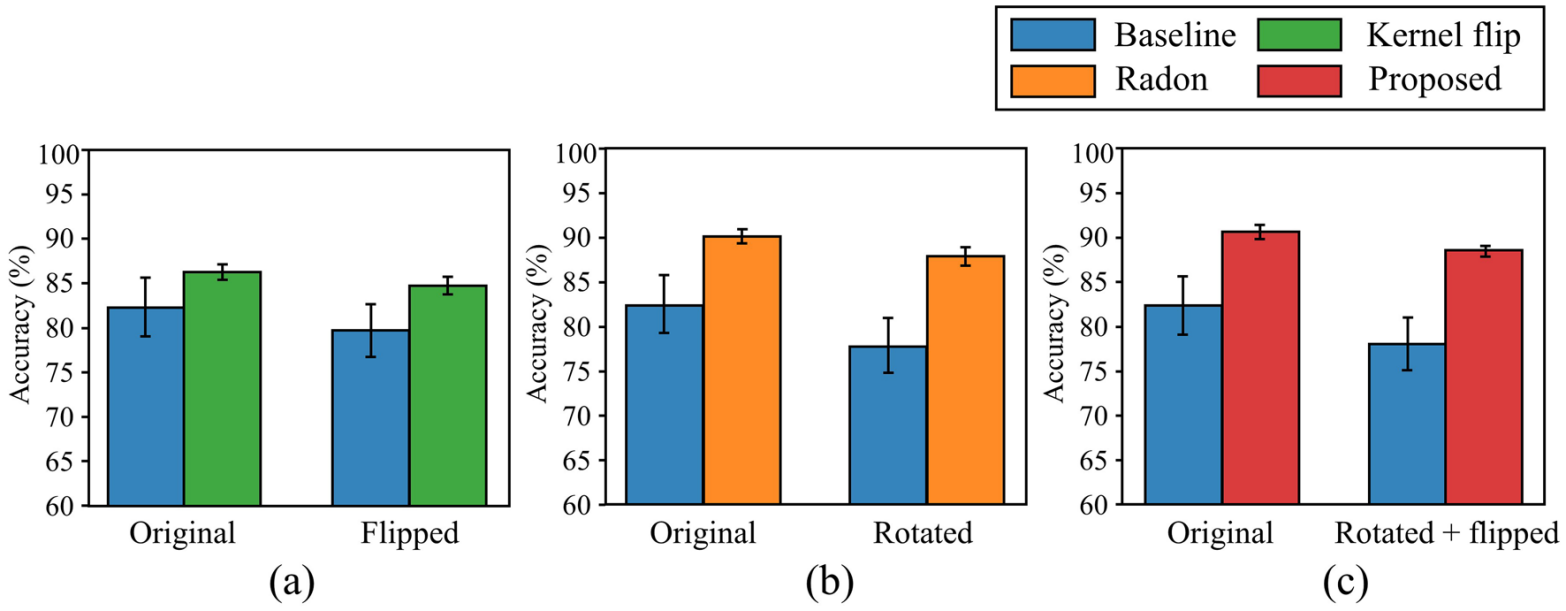
Accuracies of the comparative models for original and augmented test sets at a train set of size 6400. (**a**) Baseline and kernel flip models compared in the flipped augmented test set, (**b**) baseline and Radon models compared in the rotated augmented test set, and (**c**) baseline and proposed models compared in rotated and flipped augmented test set.

Figure 7 shows the classification accuracy of comparative models for the original and unseen augmented situations at a train set of size 6400. Figure 7a depicts the evaluation result for the original test set and flip augmented test set of both baseline and kernel flip models, Fig. 7b depicts the evaluation result for the rotation augmented test set of both baseline and Radon models, and Fig. 7c depicts the evaluation result for the rotation and flip augmented test set of both baseline and proposed models. As illustrated in Fig. 7, the Radon, kernel flip, and proposed models all achieve increased accuracy over the baseline model in each augmented test set. However, in all three cases, the accuracies are slightly decreased between two situations. It is noteworthy that the reduction gap between the baseline models is larger than that of other comparative models. This can be interpreted as the proposed model having a higher resistance to performance degradation in the generalization performance at an unseen augmented situations.

**Figure 8:**
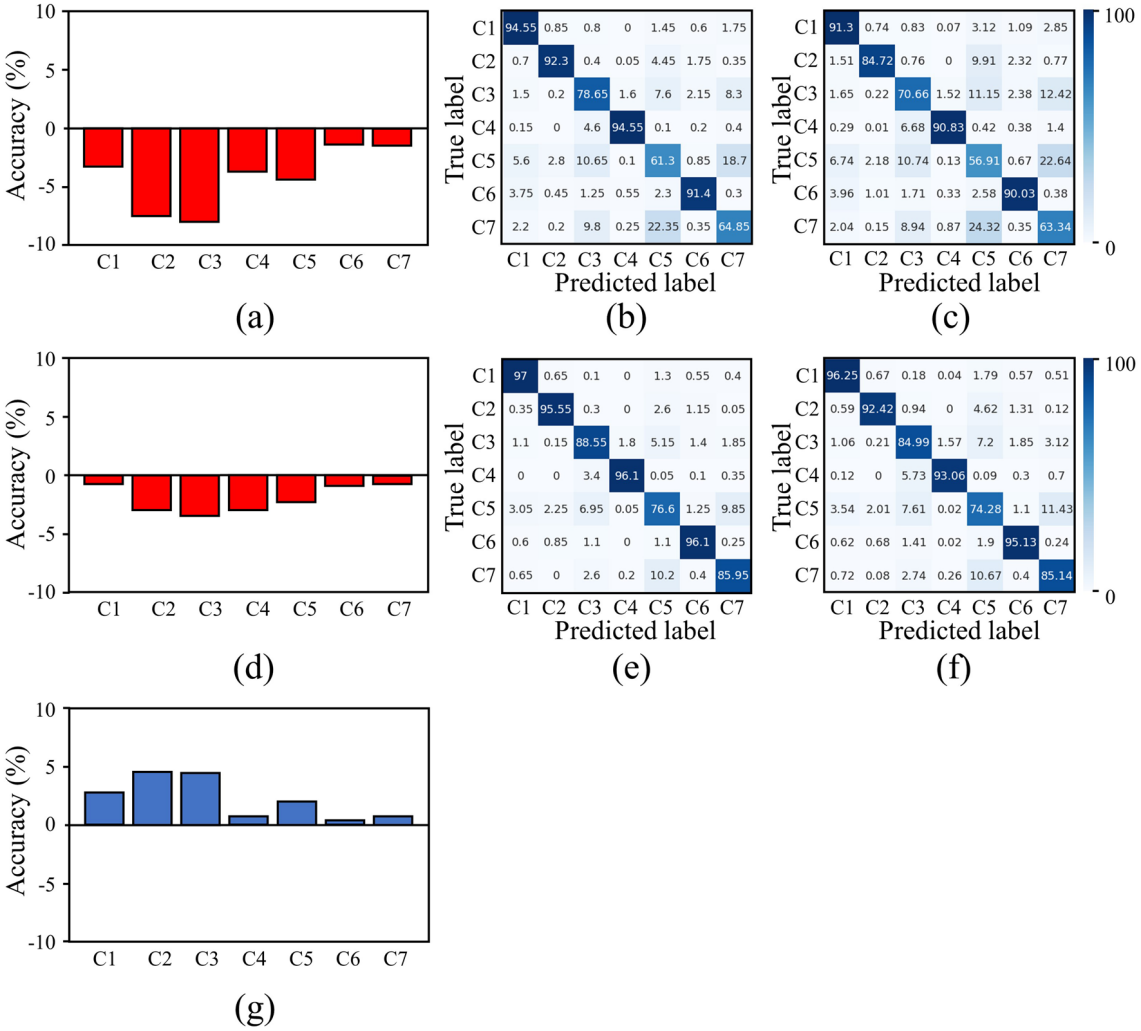
(**a**) Class accuracy gap between the (**b**) original test set and (**c**) rotated and flipped augmented test set for the baseline model at a train set of size 6400. (**d**) Class accuracy gap between the (**e**) original test set and (**f**) rotated and flipped augmented test set for the proposed model at a train set of size 6400. (**g**) Difference between (**d**) and (**a**), indicating increased generalization performance by the proposed model compared to the baseline model for each class.

Figure 8 compares the generalization performances for each class between the proposed and baseline models on a train set of size 6400. Figure 8a shows the difference in the class accuracy of the baseline models presented in Fig. 8b (the original test set) and Fig. 8c (the rotated and flipped augmented test set). Figure 8d shows the difference in the class accuracy difference between Fig. 8e (the original test set) and Fig. 8f (the rotated and flipped augmented test set) for the proposed model. Figure 8g shows the difference between Fig. 8d and Fig. 8a, which demonstrates that the proposed model has better generalization than the baseline model for each class. From Fig. 8d, we can see that the proposed model has a higher resistance to performance degradation in terms of generalization for an unseen augmented dataset for all the classes, while the classes C3 (edge-loc), C5 (loc), and C7 (scratch) show a significant increase. This extraordinary generalization performance for rotation and flip sensitive classes demonstrates that the proposed model effectively preserves the rotation and flip invariance. Additionally, this trend is in accordance with the findings of the original test set discussed in "Classification performance comparison”.

## Conclusion

In this paper, we introduce a novel method for achieving rotation and flip invariance in wafer map defect pattern classification, utilizing a combination of Radon transform and kernel flip techniques. The Radon feature ensures rotation invariance by transforming the original wafer map rotation into translation, while the kernel flipping approach provides flip invariance. Our proposed method employs an efficient network structure with a minimal number of flipped kernel branches by appropriately combining these two modules. We validate our model extensively using the WM-811K dataset with both qualitative and quantitative evaluations. Our proposed model's interpretability is demonstrated by verifying its decisions using the newly suggested multi-branch LRP method. The proposed model achieves high detection performance, even in limited data situations, by successfully ensuring rotation and flip invariance. Additionally, we assessed the proposed method's generalization performance regarding rotation and flip invariants on out-of-distribution data by using rotation and flip augmented test sets. Our study provides an efficient end-to-end deep learning model that appropriately reflects the characteristics of wafer labeling and can serve as a suitable baseline for wafer diagnosis in the future.

## Data Availability

The datasets generated during and/or analysed during the current study are available in the MIR Corpora repository (online: http://mirlab.org/dataSet/public/).
